# Supplementary material for: Mechanical forces across compartments coordinate cell shape and fate transitions to generate tissue architecture
Source: Nat Cell Biol. 2024 Feb 1;26(2):207–18. doi: 10.1038/s41556-023-01332-4 (PMC10866703; doi:10.1038/s41556-023-01332-4)
Supplement: Supplementary file 1 — Supplementary video legends (Videos 1–8). [file 41556_2023_1332_MOESM1_ESM.pdf]

nature cell biology

Article https://doi.org/10.1038/s41556-023-01332-4

Mechanical forces across compartments coordinate cell shape and fate transitions to generate tissue architecture

In the format provided by the authors and unedited

Supplementary Videos

Supplementary Movie 1. Whole embryo live imaging of basal epidermis and corresponding PIV analysis at E14.5
E14.5 whole embryo live imaging of membrane-labeled basal epidermis (left) and the corresponding PIV vectors (green arrows; right panel). Acquisitions were performed with a 40X air objective at rate of 10 min/frame for 3h 50 min. Scale bars, 20 μm.

Supplementary Movie 2. Whole embryo live imaging of basal epidermis and corresponding PIV analysis at E15.5
E15.5 whole embryo live imaging of Membrane-targeted Tomato-labeled basal epidermis (left) and the corresponding PIV vectors (green arrows; right panel). Acquisitions were performed with a 40X air objective at a rate of 10 min/frame for 2h 30 min. Scale bars 20 μm.

Supplementary Movie 3. Optical cross section of whole embryo live imaging of basal epidermis and corresponding PIV analysis at E15.5
Optical side view of E15.5 whole embryo live imaging of Membrane-targeted Tomato-labeled basal epidermis (top), with corresponding PIV vectors (middle) and strain rate magnitudes (bottom panel). Acquisitions were performed with a 40X water-immersion objective at a rate of 10 min/frame for 50 min. Scale bars 20 μm. Red crosses indicate mask used to exclude the dermal compartment from the PIV analysis.

Supplementary Movie 4. Laser ablation of E14.5 epidermis indicates tensile stress at the placode-epidermis boundary.
E14.5 whole embryo live imaging of Membrane-targeted Tomato-labeled interfollicular epidermis (top left panel) and placode (top right panel) and PIV vectors showing recoil magnitude and direction after ablation (yellow arrows) with regions of laser ablation highlighted (red circles) in the interfollicular epidermis (bottom left panel) and at the placode-epidermis boundary (bottom right panel). Images were captured every 1 s for 40 s. Ablation done at t=5 s. Scale bar 20 μm.

Supplementary Movie 5. Laser ablation of E14.5 epidermis shows lower tension within placode compared to interfollicular epidermis.

E14.5 whole embryo live imaging of Membrane-targeted Tomato-labeled interfollicular epidermis (top left panel) and placode (top right panel) and PIV vectors showing recoil magnitude and direction after ablation (yellow arrows) with regions of laser ablation highlighted (red circles) in the interfollicular epidermis (bottom left panel) and within the placode (bottom right panel). Images were captured every 1 s for 40 s. Ablation done at t=5 s. Scale bar 20 μm.

Supplementary Movie 6. Laser ablation of the fibroblast ring surrounding the placode at E15.5.

E15.5 whole embryo live imaging of Membrane-targeted Tomato-labeled placode and fibroblasts surrounding the placode (left panel) with regions of laser ablation highlighted (red rectangle) and PIV vectors showing recoil magnitude and direction after ablation (right panel; yellow arrows). Images were captured every 1 s for 40 s. Ablation done at t=5 s. Scale bar 20 μm.

Supplementary Movie 7. Cell divisions in E14.5 epidermis and placodes

E14.5 whole embryo live imaging of Histone2B-mCherry/membrane-EGFP labeled basal epidermis with placode (white circle) and cell divisions (white asterisks) highlighted. Acquisitions were performed with a 40X water-immersion objective, with a rate of 10 min/frame for 80 min. Scale bars 50 μm.

Supplementary Movie 8. Cell divisions in E15.5 epidermis and placodes

E15.5 whole embryo live imaging of Histone2B-mCherry/membrane-EGFP labeled basal epidermis with placode (white circle) and cell divisions (white asterisks) highlighted. A zoom-in of the placode base (located 8 μm below the basal epidermis Z-plane) is shown (left corner). Acquisitions were performed with a 40X water-immersion objective at a rate of 10 min/frame for 80 min. Scale bars 50 μm.
