# Supplementary material for: Mechanical forces across compartments coordinate cell shape and fate transitions to generate tissue architecture
Source: Nat Cell Biol. 2024 Feb 1;26(2):207–18. doi: 10.1038/s41556-023-01332-4 (PMC10866703; doi:10.1038/s41556-023-01332-4)
Supplement: Supplementary file 2 — Reporting Summary [file 41556_2023_1332_MOESM2_ESM.pdf]

nature portfolio

Corresponding author(s): Sara A. Wickström, M. Lisa Manning

Last updated by author(s): Nov 26, 2023

Reporting Summary

Nature Portfolio wishes to improve the reproducibility of the work that we publish. This form provides structure for consistency and transparency in reporting. For further information on Nature Portfolio policies, see our Editorial Policies and the Editorial Policy Checklist.

Statistics

For all statistical analyses, confirm that the following items are present in the figure legend, table legend, main text, or Methods section.

n/a	Confirmed	
☐	☒	The exact sample size (n) for each experimental group/condition, given as a discrete number and unit of measurement
☐	☒	A statement on whether measurements were taken from distinct samples or whether the same sample was measured repeatedly
☐	☒	The statistical test(s) used AND whether they are one- or two-sided<br>*Only common tests should be described solely by name; describe more complex techniques in the Methods section.*
☒	☐	A description of all covariates tested
☐	☒	A description of any assumptions or corrections, such as tests of normality and adjustment for multiple comparisons
☐	☒	A full description of the statistical parameters including central tendency (e.g. means) or other basic estimates (e.g. regression coefficient) AND variation (e.g. standard deviation) or associated estimates of uncertainty (e.g. confidence intervals)
☐	☒	For null hypothesis testing, the test statistic (e.g. F, t, r) with confidence intervals, effect sizes, degrees of freedom and P value noted<br>*Give P values as exact values whenever suitable.*
☒	☐	For Bayesian analysis, information on the choice of priors and Markov chain Monte Carlo settings
☒	☐	For hierarchical and complex designs, identification of the appropriate level for tests and full reporting of outcomes
☒	☐	Estimates of effect sizes (e.g. Cohen's d, Pearson's r), indicating how they were calculated

Our web collection on statistics for biologists contains articles on many of the points above.

Software and code

Policy information about availability of computer code

Data collection	Leica Application Suite X (confocal microscopy, version 2.0.0.14332)<br>Zeiss Zen software (2.3 SPI)<br>Zeiss ZEN software (blue 3.5)<br>Andor Fusion software (spinning disc confocal microscopy, version 2.3.0.44)<br>JPK SPM Control Software (version 5)<br>Nikon Software (NIS-Elements AR 5.41.01)

Data analysis	JPK Data Processing Software (Bruker Nano, version 5)<br>GraphPad Prism software (GraphPad, version 8 and 9)<br>Fiji (version 2.0.0)<br>FastQC (v0.11.9)<br>STARsolo aligner (v2.7.7a)<br>MultiQC (v1.9)<br>gseapy (v0.10.5)<br>PIVLab (v2.59)<br>Python (v3.9)<br>Tissue Analyzer (v2.3 beta2)<br>Mathematica (v12.3)<br>Cellpose (v2.0)<br>Orientation J(v2.0.5) from Fiji

For manuscripts utilizing custom algorithms or software that are central to the research but not yet described in published literature, software must be made available to editors and reviewers. We strongly encourage code deposition in a community repository (e.g. GitHub). See the Nature Portfolio guidelines for submitting code & software for further information.

Data

Policy information about availability of data

All manuscripts must include a data availability statement. This statement should provide the following information, where applicable:

- Accession codes, unique identifiers, or web links for publicly available datasets
- A description of any restrictions on data availability
- For clinical datasets or third party data, please ensure that the statement adheres to our policy

Previously published sequencing data that were re-analysed here are available under accession code GSE122043.
The vertex model is available under the following link: https://github.com/ZhangTao-SJTU/tvm
Custom-built Python and Mathemathica scripts used in the manuscript are available under the following link:https://github.com/WickstromLab
All other data supporting the findings of this study are available from the corresponding author on reasonable request.

Human research participants

Policy information about studies involving human research participants and Sex and Gender in Research.

Reporting on sex and gender	N/A
Population characteristics	N/A
Recruitment	N/A
Ethics oversight	N/A

Note that full information on the approval of the study protocol must also be provided in the manuscript.

Field-specific reporting

Please select the one below that is the best fit for your research. If you are not sure, read the appropriate sections before making your selection.

☒ Life sciences ☐ Behavioural & social sciences ☐ Ecological, evolutionary & environmental sciences

For a reference copy of the document with all sections, see nature.com/documents/nr-reporting-summary-flat.pdf

Life sciences study design

All studies must disclose on these points even when the disclosure is negative.

Sample size	Sample size was determined based on previous experience, published literature (PMID: 30063206, 28700594, 29662173) . Sample size for each experiment is indicated in figure legends.
Data exclusions	For AFM measurements outlier identification was carried out to remove rare individual measurements that represented apparent artefacts, no other data points were removed from the experiments
Replication	All experiments were performed using at least three biological replicates. Number of replicates for each experiment is indicated in the corresponding figure legend. Several steps were taken to ensure the reproducibility of experimental findings and key results were confirmed using complementary experimental approaches.

Randomization	Samples were not randomized, randomization was not relevant as samples were grouped according to genotype or treatment.
Blinding	Blinding was used where relevant (AFM analysis). It was not relevant in the in vivo studies as phenotypes of mice were clear to experimenters. Whenever possible automated software algorithms were used for unbiased quantification of cell volumes (Fig. 1g-h), cellular and tissue morphology (Extended Data Figure 1b-c; Extended Data Figure 2a, b), PIV analysis and strain rate extraction (Fig.2a-d and i; Extended Data Figure 2c-e, h, i) staining intensities (Fig. 2e-g; Fig.4f, g, j-m; Fig. 6b, c; Extended Data Figure 2f,g; Extended Data Figure 4b-d; Extended Data Figure 5h,i o, p; Extended Data Figure 6a,b) , fiber alignment (Fig.2h) and sequencing data (Extended Data Figure 6f).

Reporting for specific materials, systems and methods

We require information from authors about some types of materials, experimental systems and methods used in many studies. Here, indicate whether each material, system or method listed is relevant to your study. If you are not sure if a list item applies to your research, read the appropriate section before selecting a response.

Materials & experimental systems

n/a	Involved in the study	
☐	☒	Antibodies
☒	☐	Eukaryotic cell lines
☒	☐	Palaeontology and archaeology
☐	☒	Animals and other organisms
☒	☐	Clinical data
☒	☐	Dual use research of concern

Methods

n/a	Involved in the study	
☒	☐	ChIP-seq
☒	☐	Flow cytometry
☒	☐	MRI-based neuroimaging

Antibodies

Antibodies used	goat anti Edar (R&D Systems AF745; 1:200), rabbit anti-p-MLC2 (p-Ser20; Abcam 2480; 1:100; 1:100), rabbit anti-Vimentin (Abcam 185030; 1:100), rabbit anti-Sox9 (Cell Signalling, 82630; 1:100), rabbit anti-Sox2 (Millipore Sigma, AB5603; 1:200), mouse anti-Twist2 (Abcam, 50887; 1:200), mouse anti-Ki67 (Cell Signaling, 9449; 1:300), rabbit anti-active YAP1 (Abcam, 205270; 1:100), guinea-pig anti-Keratin 14 (Progen, GP-CK14; 1:300), rabbit anti-Collagen IV (Abcam, 6586; 1:200), rabbit anti-Laminin 332 (gift from R.E Burgeson; 43; 1:20 000), rat anti-Laminin a5 (504 44; self-produced; gift from L. Sorokin; 1:20 000), chicken anti-beta-galactosidase (Abcam, 9361; 1:500). F-actin was detected using Alexa647-conjugated phalloidin (1:500).<br>The following secondaries (all from Invitrogen): anti-rabbit IgG Alexa Fluor 488 / 568 (A11008 / A10042; 1/500); anti-mouse IgG1 / IgG2A Alexa Fluor 546 (A21123 / A21134; 1/500); anti-rat IgG-rat Alexa Fluor 647 (A21247; 1/500); anti-goat IgG Alexa Fluor 488 (A21467) and anti-chicken IgG Alexa Fluor 488 (A21449; 1/500) were used to visualize primary antibodies.
Validation	All antibodies are well characterized and widely used in the literature. They were applied according to datasheet instructions or previously published protocols. Antibodies were additionally validated as follows:<br>- Inhibitor studies followed by immunofluorescence on primary mouse keratinocytes (YAP, Extended Data Figure 6a, b) or immunofluorescence on mouse tissues (Ki67, Extended Data Figure 6c, d), or zebrafish tissues (pMLC2; PMID: 33208950).<br>- Immunofluorescence of mouse tissue from knockout or transgenic mouse models (Laminin alpha5 (PMID: 27234307), beta galactosidase (Extended Data Figure 1a, b and PMID 30063206)).<br>- Immunofluorescence on mouse tissue followed by observing expected pattern of histological staining (as described in the literature for example PMID: 26256211, PMC6361530 and 30887615) and colocalization with additional markers of same state or process or specific morphology: (Keratin 14, localization in the basal layer of the epidermis, Extended Data Figure 4h ; Twist2 and Vimentin, specific expression in fibroblasts, Extended Data Figure 5a; Collagen IV and Laminin 332, localisation specifically at the basement membrane Fig.6b; Sox9, localisation specifically in placode cells, Fig.4f and PMID29662173; Sox2, localisation specifically in the dermal condensate, Extended Data Figures 4e and 5c and PMID 30063206; Edar, enrichment specifically in placode cells, Fig.1b and Fig.2h and PMID: 30887615).<br>.

Animals and other research organisms

Policy information about studies involving animals; ARRIVE guidelines recommended for reporting animal research, and Sex and Gender in Research

Laboratory animals	Wild-type C57Bl6J mice, Myh9 floxed mice (obtained from the European Mouse Mutant Archive; EM:02572) were crossed with the K14-Cre line (Hafner et al., or the Twist2-Cre line obtained from JAX laboratories (stock #008712). Membrane-targeted Tomato reporter mice (R26RmT/mG) were from JAX laboratories (stock #007676). Histone2B -mCherry/membrane-EGFP (R26R-RG mice; were from Riken Laboratories for Animal Resources and Genetic Engineering (LARGE) and were crossed with K14-Cre line to obtain epidermis-specific expression. FGF20-LacZ reporter mice have been previously described in Huh et al., Genes Dev 2013.
Wild animals	Study did not involve wild animals
Reporting on sex	Studies were carried out on embryos of both sexes as no sex-specific difference relevant for the processes studied here were noted.

Field-collected samples	Study did not involve samples collected from the field
Ethics oversight	All mouse studies were approved and carried out in accordance with the guidelines of the Finnish national animal experimentation board (ELLA) or the Ministry for Environment, Agriculture, Conservation and Consumer Protection of the State of North Rhine-Westphalia (LANUW), Germany.

Note that full information on the approval of the study protocol must also be provided in the manuscript.
